# Multimodal OCT Reflectivity Analysis of the Cystoid Spaces in Cystoid Macular Edema

**DOI:** 10.1155/2019/7835372

**Published:** 2019-03-20

**Authors:** Roberta Farci, Alexandre Sellam, Florence Coscas, Gabriel J. Coscas, Giacomo Diaz, Pietro Emanuele Napoli, Eric Souied, Maria Silvana Galantuomo, Maurizio Fossarello

**Affiliations:** ^1^Eye Clinic, University of Cagliari, Cagliari, Italy; ^2^Eye Clinic, Hôpital Intercommunal, Créteil, France; ^3^Biomedical Science Department, University of Cagliari, Cagliari, Italy

## Abstract

**Purpose:**

To compare and evaluate images of macular cysts with different degrees of reflectivity (from gray to black signal) as observed in B scan spectral domain OCT (SDOCT) and EnFace OCT with decorrelation signal obtained with OCT-angiography (OCTA) in eyes with cystoid macular edema (CME) secondary to diabetic retinopathy (DR) and retinal vein occlusion (RVO).

**Methods:**

Images from 3033 patients affected by CME secondary to diabetes or RVO examined OCTA (Optovue XR Avanti, Optovue, USA) at the University Eye Clinic of Créteil, Hôpital Intercommunal, France, and at the University Eye Clinic of Cagliari, “San Giovanni di Dio” Hospital, Italy, were retrospectively examined. The deep capillary plexus OCTA images and the corresponding EnFace OCT images, both acquired with the same automatic segmentation, had been overlapped to compose RGB color images as red and green channels, respectively, using ImageJ software (National Institutes of Health, Bethesda, MD). Afterward, linear regions of interest were traced on the color images to obtain the profiles of OCTA and EnFace gray values. Number of pixels, mean gray value and standard deviation of the area traced in OCT-A, and EnFace image were analyzed and statistically correlated. Data were exported to Excel to create the plots.

**Results:**

94 patients with DME and 27 patients with RVO showed intraretinal macular cystoid spaces with similar homogeneous, gray-looking content; 73 patients with DME and 113 patients with RVO showed macular cystoid spaces with homogeneous, black-looking content, as observed at SD-OCT, EnFace and OCTA scans. Interestingly, the limits of macular cystoid spaces were clearly detectable with OCTA. The analysis of red and green profiles demonstrated a clearly visible overlap between average OCTA and EnFace signal observed around cystoid spaces that could be attributed to a relationship between the dynamic vascularization and the structural density of the tissue.

**Conclusions:**

This is the first investigation that characterizes and correlates OCTA and EnFace signals on images of macular cystoid spaces in DR and RVO. The low intensity OCTA signals observed inside cystoid spaces raise a relevant question about their nature, as to whether they are due to the presence of corpusculated material pouring out from bloodocular-barrier or they should be considered OCTA artifacts.

## 1. Introduction

Macular edema (ME) is the consequence of many ocular diseases such as diabetic retinopathy (DR), retinal vein occlusion (RVO), and uveitis and is a major cause of visual loss [1–9].

The pathophysiology of ME is not fully understood yet, but the breakdown of inner and/or outer blood–retina barriers (BRB) is a key event in the onset of the ME [10–14].

Although for a long time clinical examination has been considered the gold standard for the diagnosis of ME [4], nowadays, optical coherence tomography (OCT) offers a better technique to detect ME with more accuracy and even before clinical appearance. In particular, spectral domain OCT (SD-OCT) allows visualization of ME in unprecedented details, so that lesions can be evaluated in all individual retinal layers including location, extension, pattern, and additional features, in an objective and standardized way [15, 16]. A number of studies analyzed the OCT characteristics of ME in different diseases [17–19], such as the presence of diffuse ME, cysts, and subretinal fluid. In diabetic macular edema (DME), SD-OCT demonstrated fluid-containing ovoid cavities (cystoid spaces or pseudocysts, at times inappropriately called cysts) of different size, separated by highly reflective septa, supposed to be represented by Muller cells axons [20]. The cavities appear located mainly in the INL and OPL, as confirmed by histological studies [21, 22].

Four different types of macular cysts have been described using SD-OCT [23, 24]: low reflective cysts, cysts with heterogeneous reflectivity, cysts with hyperreflective foci associated significantly with heterogeneity or higher levels of reflectivity, and solid-appearing cysts. The different values of reflectivity of the cystoid spaces presumably reflect the nature of their content, either plasma, blood, hyaline or fibrinous material, or macrophages, and implicate different types of vascular hyperpermeability.

With the advent of OCT-angiography (OCT-A), a new, noninvasive technique capable of detecting the flow of red blood cells (RBC) inside microvessels without intravenous contrast agent injection, it is now possible to visualize retinal microcirculation directly, in a multilayer, tridimensional way [25–29]. OCT-A provides volumetric data and shows both retinal structure and blood flow information in tandem. The erythrocytes flow inside blood vessels is rendered on OCT angiograms by white flow pixels delineating a vascular network [10]. Moreover, the device displays a stationary EnFace OCT (EF-OCT) of the same angio image and a structural OCT. Therefore, this technique permits visualizing the majority of clinically relevant vascular changes characterizing DR [30, 31] and RVO [8, 32, 33]. 

OCTA also provides quantitative data about flow area, nonflow area, and vascular density [34].

Numerous OCT-angiography algorithms have recently been proposed for the purpose of distinguishing between stationary and nonstationary tissue and for contrasting microvascular networks, including optical microangiography (OMAG), speckle variance, phase variance, split-spectrum amplitude decorrelation angiography (SSADA), and correlation mapping (see Anqi Zhang et al., for a review) [16, 25].

In particular, the SSADA algorithm identifies blood flow by calculating the decorrelation of signal amplitude from consecutive B-scans performed at the same retinal acquisition level [25]. Surprisingly, in some cases of CME this algorithm has highlighted a decorrelation signal, i.e., a signal of RBC flow, in correspondence of intraretinal cystoid spaces [33, 34].

However, the detection of a decorrelation signal outside vascular structures cannot be easily attributed to erythrocyte movements, and it has been interpreted in terms of Brownian movements of the cystoid content and hence classified as an artifact [35]. It is well known that image artifacts are common in OCT-A images, due to motion error and inappropriate software correction, including poor recognition of the background noise.

OCTA signals are generated by detecting motion between repeated B scans using decorrelation or fluctuations on a pixel by pixel basis. Movement of the patient's eye, head, or body results in widespread decorrelation over the entire B scan. Reflections from retinal features or pathology could produce false OCTA flow signals because of variations in OCT signals which are unrelated to blood flow within the pathology itself.

Therefore, in this study we try to clarify the nature of SSADA intracystic signal observed in CME, associated with DR and RVO, through a quantitative comparison between stationary EF-OCT and OCT-A orthoplane images realized by the AngioVue Imaging System (Optovue, Inc., Freemont, CA) [10].

The rationale is that even if the decorrelation signal does not always match the reflectivity signal recorded across the edematous vacuoles, it may nonetheless represent a “real” signal, originating from material present inside the cystoid spaces, and, therefore, it should not be considered as an artifact, strictly speaking.

## 2. Material and Methods

### 2.1. Patients Characteristics

Participants of the present study were previously enrolled in this cross-sectional study October 2014 and January 2016 at two centers, the Department of Ophthalmology at the Hôpital intercommunal de Cr.teil (Cr.teil, France) and Eye Clinic of San Giovanni di Dio Hospital of Cagliari (Cagliari, Italy). Eligible subjects were male and female with cystoid macular edema due to diabetic retinopathy (DR) and retinal vein occlusions (RVO). They were selected from a database of 3033 patients that underwent OCT-A examination with the AngioVue Imaging System (Optovue XR Avanti, Optovue, Inc., Freemont). Inclusion criteria were as follows: (1) age ≥18 and <95 years; (3) visual acuity ≥+0.4 LogMAR units in the study eye; and (4) subjects likely to attend follow-up visits during the study period. The main exclusion criteria were as follows: (1) CNV in one or both eyes; (2) age related macular degeneration; (3) progressive ocular diseases (severe glaucoma or other severe retinopathies); (4) corneal or lens opacities precluding retinal evaluation; (5) retinal pathologies for reasons other than AMD; and (13) subjects not covered by French and Italian National Health Systems.

They underwent OCT-A examination with the AngioVue Imaging System (Optovue XR Avanti, Optovue, Inc., Freemont, CA, USA) (Optovue XR) at the University Eye Clinic of Créteil, Paris, France, and at the University Eye Clinic of Cagliari, Italy, between October 2014 and January 2016. Collected data included a complete eye examination consisting of slit lamp, indirect ophthalmoscopy, fluorescein angiography, and SD-OCT (HRA/Spectralis, Version 5.4.4.0; Heidelberg Engineering, Germany, or Cirrus, Zeiss). For the selection of patients, CME was defined as the presence of intraretinal cystoid, like spaces displaying a zero reflectivity (black cystoid spaces), or a detectable gray reflectivity, as observed on SDOCT images. Only images of adequate quality were considered.

### 2.2. Imaging and Grading Methods

For each eye, we analyzed deep capillary plexus Optovue images, visualized with automated segmentation. We obtained a pair of OCT-A and EF-OCT 8-bit images, aligned on the level and embracing an area of 3x3 mm, with a resolution of 3 *μ*m/pixels. The pair of OCT-A and EF-OCT images were then imported into ImageJ (Fiji) program [35] for image processing. Angiograms of the superficial capillary plexus (SCP) and deep capillary plexus (DCP) were automatically segmented using preset parameters. The automated segmentation of DCP was used to measure the capillary density. Angioflow density was calculated for DCP using AngioAnalytics software. To determine the percentage of the Angioflow surface on the complete surface of the angiogram, we utilized the color coded scale. For quantitative estimations, the perimeter of the cysts was manually traced by using Image J software, and measurements of the mean level of gray and the count of gray pixels (area) of the cystoid region were performed on EF-OCT; subsequently, the previously drawn cyst perimeter was dragged onto OCT-A images, and similarly the mean gray level was calculated (Gray Histogram, ImageJ plug in). The average gray value outside the cyst was also evaluated on both OCT-A and EF images. The value inside the cysts was then subtracted from the value outside the cysts, to normalize against brightness and contrast changes made by the operator during the clinical examination to optimize the visual appearance of images. Data were then imported into Excel spreadsheet for statistical analysis (Principal Component Analysis). The reflectivity signal (EnFace) and the decorrelation signal (OCT-A) grayscale images were imported into Fiji-ImageJ37 and merged into a single 24- bit RGB color image, setting the reflectivity signal and the decorrelation signal as the green and red channel, respectively. The original gray values of EF-OCT and OCT-A images were entirely saved without changes in RGB images. By doing so, we obtained a pair of reflectivity and decorrelation OCT-A values for each pixel of the RGB image (Figures 3 and 4). A color composition of a pair of OCT-A and EF-OCT images is shown in Figure 2. Data were then exported to Excel spreadsheet for graphical and statistical processing. Optovue OCT-A is capable of detecting capillary flow density in the macular region (Figure 6).

### 2.3. Statistical Analysis

Data of OCT-A and EnFace images were processed by principal component analysis. This is a basic multivariate exploratory method generally used to summarize the information (i.e., variance) of multiple variables into one or two conventional variables called principal components. In the present study, more than 90% of the total variance of OCT-A and EnFace images could be summarized by the first principal component.

Number of pixels, mean gray value, and standard deviation of the area traced in OCT-A and EnFace image were analyzed and statistically correlated. Statistical analysis was performed via computer by using Statistical Package for Social Science SPSS version 21.0 (SPSS, Inc., Chicago, IL, USA). Mean and standard deviation were calculated for each OCT-A parameter. Data distribution was evaluated by Shapiro-Wilk test and Kolmogorov Smirnov test with Lilliefors correction. Correlation analysis between OCT-A parameters was performed by using Spearman's *ρ* test. The sample size calculation was performed assuming an alpha risk of 0.05 and a beta risk of 0.2 in a two-sided test. To recognize as statistically significant a correlation coefficient greater than or equal to 0.30 would require 84 cases. The p values less than 0.05 were considered significant.

## 3. Results

We found a total of 201 patients (232 eyes) affected by CME (6,63%) with either black or gray cystoid spaces as assessed by OCT-B scan: 137 patients, 96 males and 41 females (168 eyes), were affected by DR; mean age of males was 64.28 years (D.S. Å}7.33); mean age of females was 67.70 years (D.S. Å}6.98) and 64 by OVR (76 eyes) 22 males (mean age: 59.43 years D.S. Å}7.33) and 42 females (mean age: 57.34 years D.S. Å}9.94) (Table 1).

In B scan OCT images, we observed totally black cystoid spaces in 163 eyes, gray cystoid spaces in 69 eyes, and cystoid spaces with a black and white gravitational pattern (a black upper zone and a white lower zone) in 4 eyes (Figure 5). Mean permanence time of cystoid spaces was 5.34Å}7.47 months; the follow-up duration was 27.64Å}21.98 months. Mean vertical diameters measures were black cysts 348.384 Å} 149.341 *μ*m; gray cysts 351.759 Å} 147.835 *μ*m; mixed cysts 346.480 Å} 148.139 *μ*m. Mean horizontal diameters are black cysts 366.624 Å} 177.353 *μ*m; gray cysts 366.849 Å} 174.505 *μ*m; mixed cysts 364.324 Å} 176.009 *μ*m (Table 1).

Cystoid walls and the gray content are also visible in correspondence of the avascular foveal zone of the superficial capillary plexus.

For EF-OCT and OCT-A analysis we considered only gray cystoid spaces, selected among 69 eyes of 54 patients (mean age: mean 70.04Å) (12.59 years) affected by CME due to DR (57 eyes of 42 patients, 16 females and 26 males; mean age: 61.65Å)5.44; mean age of females: 76.6 yrs Å} and 15.69 DS; mean age of males: 66.4 yrs Å}12.16) or to RVO (12 eyes of 12 patients), mean age: 69,85Å}25,24 yrs; 8 females (mean age: 85.4 yrs Å)3.24 SD) and 4 males (mean age: 61.5 yrs Å)13.5 SD) (Table 2).

**Figure 1:**
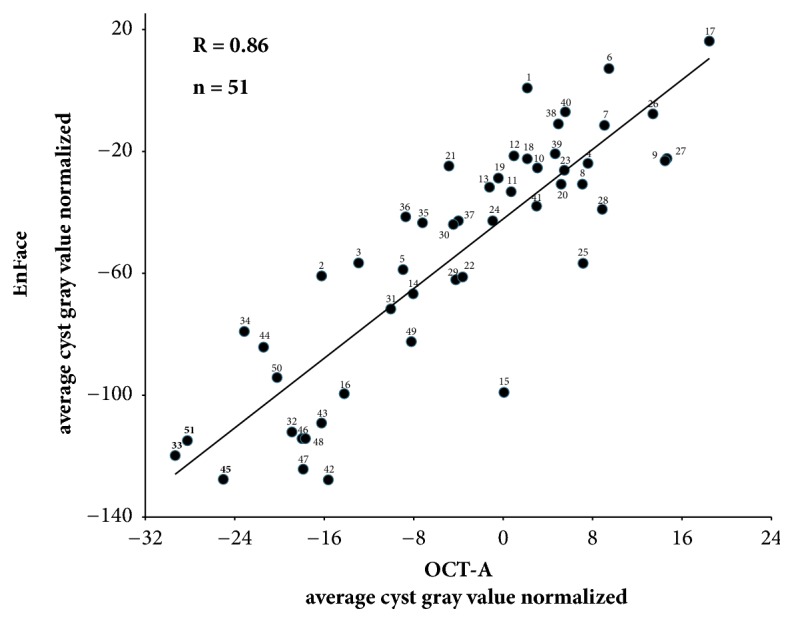
Gray values inside the cysts were subtracted from the values outside the cysts, to normalize against brightness and contrast changes made by the operator to optimize the visual appearance of images. This procedure showed a strong correlation between average OCT-A and EnFace gray values of cysts (*ρ* = 0.86).

**Figure 2:**
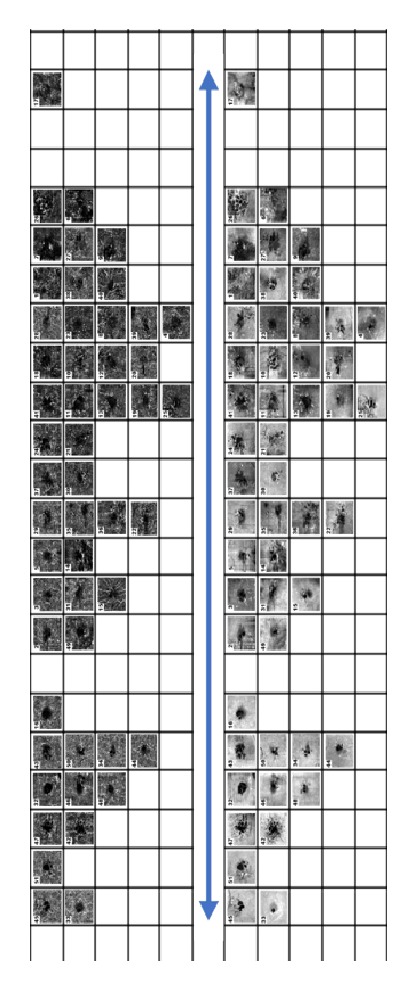
Merging of OCT-A and EnFace images into a color RGB image.

All cystoid spaces showing variable gray levels in B scan images showed a detectable level of gray also in EF-OCT and OCT-A images. The mean gray level observed was 20,86 (Å) ±18.32 SD in EF-OCT images and 15.29 (Å) ±8.15 SD in OCT-A images, and the data were statistically correlated (*ρ* =0.374). A statistically significant correlation was also observed between the mean gray level and the area of the cysts (*ρ*=0.267): the larger was the cystoid space, the higher was the mean gray value. Moreover, a statistically significant correlation between OCT-A and EnFace signals was found at the level of the cystoid spaces (Figure 1), after normalization against brightness and contrast changes made by the operator during the clinical examination (R=0.86). In the plot of Figure 1 values are represented by a collage of the original (not normalized) OCT-A (upper panel) and EnFace (lower panel) images. Principal Component Analysis indicated that the trendline summarizes nearly 93.21% of the total variance of all OCT-A and EF- OCT cysts data, taken together. In view of this result, the trendline provides a grading that may be adopted for a linear grading of the severity of the pathology.

**Figure 3:**
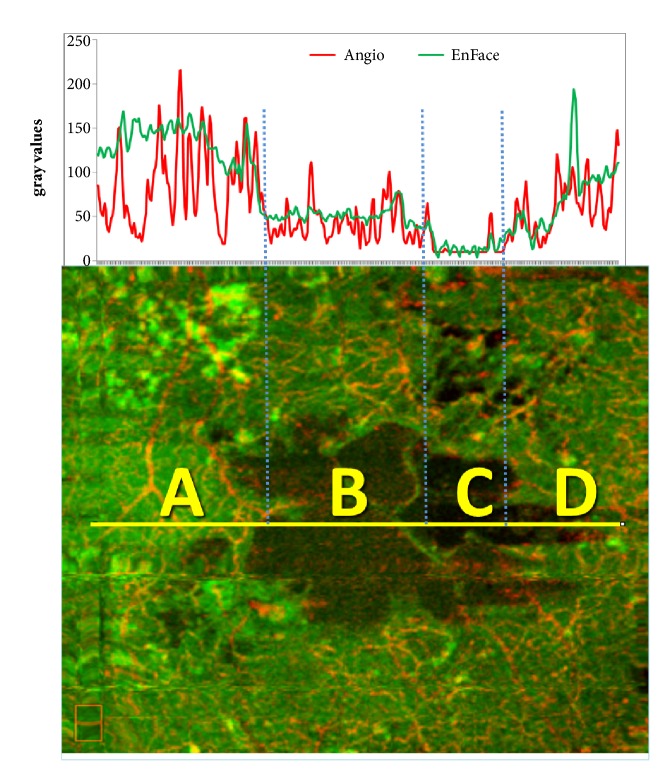
The color image makes it possible to correlate OCT-A and EnFace values on any arbitrary line (linear region of interest). This example shows a line that crosses a cyst through 4 regions. Segment A intercepts the tissue surrounding the cyst, B and C indicate two portions, one pale and one dark, of the cyst, and D highlights an area where cystoid space mixes with tissue. Out of the cyst (A) the plot shows sharp OCT-A peaks associated with high but smoother EnFace values. A similar relationship, although at a lower intensity level, is present also inside the cyst, in particular in the B tract, where small, but significant OCT-A peaks correlate with low EnFace levels. In the C tract both OCT-A and EnFace reach the lowest intensity levels, near to the image background signal. In D we can observe mixed peaks, according to the different structures present in this segment.

**Figure 4:**
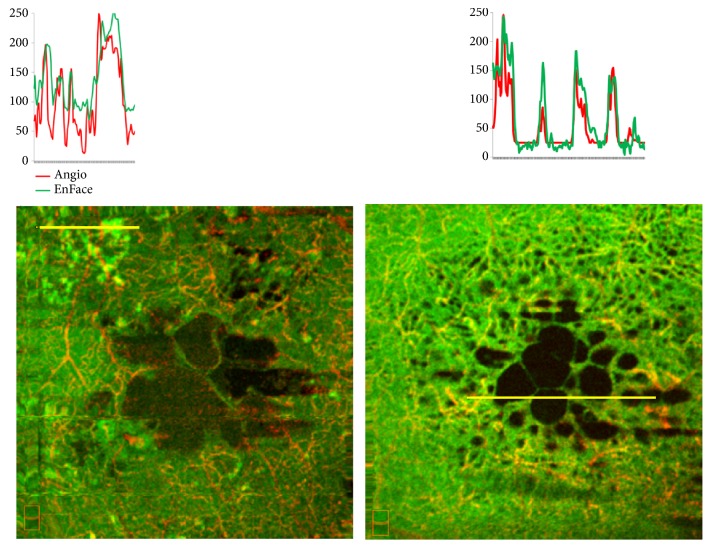
The correlation between OCT-A and EnFace values is also present in regions far from the cysts, which display the highest OCT-A and EnFace values (left panel), as well as in the thin septa interspersed with dark cysts (right panel).

**Figure 5:**
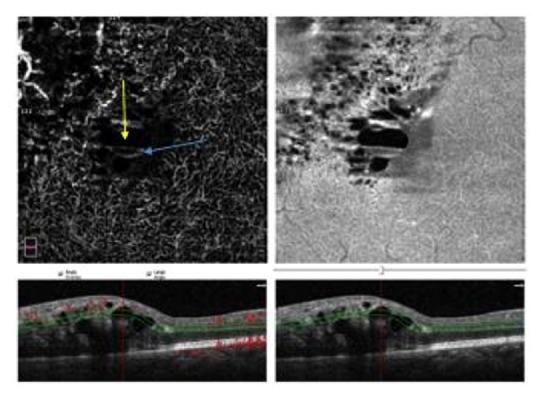
An example of gravitational level seen as a gray part sit in the lower part of the cyst (indicated by the blue arrow) and the black zone, located in the upper part of the cystoid space (indicated by the yellow arrow).

**Figure 6:**
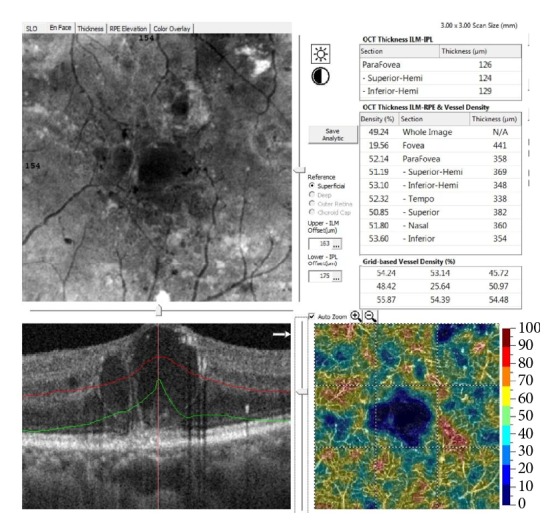
Capillary flow density of Optovue OCTA.

Capillary flow density values were calculated by means of the manual segmentation in correspondence of the deep capillary plexus. The value of mean capillary flow density inside the gray cystoid spaces was 21.03% (+/- 1.62 SD), and inside the black cystoid spaces it was 24.17% (+/ 0.28 SD). However, in the septa and in the tissue around the cystoid spaces, the observed values were about 40% for gray cystoid spaces and about 55% for black cystoid spaces (according to the color coded scale). A statistically significant correlation between the reflectivity signal (EF-OCT) and the decorrelation signal (OCT-A) was also found on 24-bit RGB color images at the level of cystoid spaces and the tissue surrounding the cysts (Figures 3 and 4). On this image, a line has been traced that crosses 4 areas, indicated by letters A-D: the tissue surrounding a cyst (A), two distinct portions of the cyst, one pale and one dark (B, C) and one mixed area (D). The plot of OCT-A and EnFace values found along this linear region of interest (ROI) is reported on the top of the image.

Out of the cyst (A) the plot shows sharp OCT-A peaks associated with high, even though smoother EnFace peaks. A similar relationship, although at reduced intensity levels, is present also inside the cyst. In particular, we could observe in (B) small but significant OCTA peaks associated with low EnFace levels, whereas in (C) both OCT-A and EnFace reach the lowest intensity levels, near to the background signal. A correlation was also found in regions of the same image with the highest OCT-A and EnFace intensities as well as in the thin septa of dark cysts. Moreover, statistical analysis permitted excluding a correlation between the residence time and the mean level of gray of cystoid spaces and between the visual acuity and the mean gray level of the cystoid spaces.

Capillary flow density analysis revealed a relative normal value around black cystoid spaces and reduced values around gray cystoid spaces and zero capillary density inside cystoid spaces.

## 4. Discussion

Optovue OCT-A utilizes a split-spectrum amplitude decorrelation angiography (SSADA) algorithm which measures the variation in reflected OCT signal amplitude between consecutive cross-sectional scans to visualize retinal blood flow [25]. Therefore, presumed stationary tissue structures such as intraretinal edematous cystoid spaces, which in B and C scan (structural OCT and EF-OCT) display variable gray signals, are not expected to elicit flow signals and should not appear in OCT-A images [25].

However, in a number of OCT-A images, we observed that cystoid spaces contain a variable, nonhomogeneous texture of gray pixels, which precisely colocalizes with the gray pixel texture observed in EF-OCT images. Moreover, when we compared the relative mean values of gray displayed by the two signals across the cystoid spaces, we found that although the reflective signal of EF-OCT images shows higher values than the decorrelation signal of OCT-A images, the relative fluctuations in intensity of the two signals correlate significantly, both within and around the cystoid spaces.

Therefore, we wondered if such an OCT-A decorrelation signal could come from materials of vascular origin, such as RBCs, proteins, or lipids which may float and produce speckles that would be detected and elaborated by the SSADA software as gray signal. In a study on cystoid spaces using B scan SD-OCT [23], Horii et al. suggested that reflective black signal would represent fluid and reflective gray signal low would represent and high weight proteins, fibrin, hyaline material, macrophages, or erythrocytes. Since EF-OCT is also based on B scan technique (C scan), we can assume that the EnFace gray signal recorded inside the cystoid spaces is similarly related to the nature of their content (fluid or solid).

By considering the strong correlation between fluctuations of EF-OCT and OCT-A signals, we may assume that also the OCT-A signal observed inside cystoid spaces originates from floating particles of vascular origin and does not represent a noise or other artifacts, resulting from flaws in software or equipment. Spaide et al. [33] suggested that a gray appearance of retinal cystoid spaces in OCT-A images may be due to a residual motion or Brownian movements of their content. Therefore, if cystoid spaces contain floating erythrocytes, which SSADA software detects and elaborates as gray signal, we cannot consider them such signal as a real artifact.

On the contrary, in the case that the floating particles are represented by other molecules crossing the BRB, like heavy weight proteins, hyaline material, or macrophages that have phagocytized lipids, the decorrelation gray signal should be considered nonselective, since it is elicited from floating molecules different from RBCs.

Finally, we cannot exclude that the OCT-A decorrelation signal contains also a stationary speckle signal. In this case, static structures or materials characterized by a sufficiently high level of OCT reflectance, such as fibrin or hard exudates located within and around the cystoid spaces, may appear in OCT-A images, as already highlighted by Kashani et al. [35] and it would represent a real artifact, being an error of the SSADA software. Nevertheless, in our paper, gray cysts do not resolve in the formation of hyperreflective material [35].

It is interesting to note that, inside the capillary empty cystoid spaces (either black or gray), the capillary flow density value of deep capillary plexus amounts approximately to 20%, even if it corresponds to a square of dark blue color (zero density). Thus, a discrepancy exists between the numeric figure and the color code scale. Areas corresponding to cystoids septae and gray content, though, present a slightly higher percentage of density, this would mean that a capillary component is located within these locations.

Concerning the OCT-A gray signal observed in correspondence of the tissue around cystoid spaces, it could conceivably arise from RBCs flow in smaller capillaries of parenchyma surrounding the cystoid wall. This possibility is supported by the observation that the capillary flow density in the tissue around the cysts has low, but significant, values, higher in black cysts and lower in gray cysts, in accordance to Man. et al's observations [34].

In conclusion, in this study we verified by image J analysis a strong correlation of EnFace OCT and OCT-A signals inside the cysts and in correspondence of the tissue neighboring cystoid walls, in images obtained from patients affected by CME secondary to DR and RVO [36]. No significant differences were observed between DR and RVO CME. While it is conceivable that the OCT-A gray signal observed in correspondence of the tissue around cystoid spaces could arise from the flow of RBCs in smaller capillaries of parenchyma and septa surrounding the cystoid wall, it is more difficult to understand the origin of the OCT-A decorrelation signal in areas void of capillaries, such as the cystoid spaces. It is likely that they contain different static or floating materials, which can elicit speckle signals, detected and elaborated by SSADA software as gray signal.

A comparison of OCT-A images obtained by Optovue with other OCT-A imaging systems employing different algorithms for flow analysis should help clarify the exact nature of signal arising from edematous cysts and other apparently stationary intraretinal structures.

## Figures and Tables

**Table 1:** Demographic characteristics of patients with cysts.

Sex	95 males,		48 males	

Age	64.28 yrs (males)		59.43 yrs (males)	

	67.70 (females)		57.34 yrs (females)	

Disease	Diabetic	Macular	Retinal	Venous
	Edema		Occlusion	

Eyes	168		64	

Permanence of cysts	5.34±12.02 months			

Vertical Diameters	374 ± 248.9 *µ*m			
	(black cyst)			
	489 ± 70			
	(gray cysts)			
	266.5 ± 61.51 *µ*m			
	(mixed cysts)			

Horizontal Diameter	354.11 ± 79.19 *µ*m			
	(black cysts)			

**Table 2:** Demographic characteristics of patients with gray cysts.

Sex	26 males and 16 females	4 males and 8 females

Age	61,65±5,44 yrs	69,85±25,24 yrs

Disease	Diabetic Macular Edema	Retinal Vein Occlusion

Eyes	57 eyes	12 eyes

## Data Availability

Authors confirm that all data underlying the findings in the present study are freely available in the manuscript.
